# Postoperative changes in pulmonary function testing and patient reported outcomes following lung resection: a review

**DOI:** 10.3389/fsurg.2026.1998046

**Published:** 2026-10-08

**Authors:** Shannon Parness, Ashton Rierson, Panagiotis Tasoudis, Benjamin Haithcock

**Affiliations:** 1 UNC School of Medicine, Chapel Hill, United States; 2 Division of Cardiothoracic Surgery, Department of Surgery, UNC School of Medicine, Chapel Hill, United States

**Keywords:** lobectomy, lung resection, non-small cell lung cancer, PFT (pulmonary function test), pneumonectomy, segmentectomy, wedge resection

## Abstract

Pulmonary resection is a primary treatment for patients with resectable non-small cell lung cancer, but removal of lung tissue can result in significant changes in postoperative pulmonary function and patient recovery. This review examines changes in pulmonary function tests (PFTs) and patient-reported outcomes following lung resection, with consideration of differences among surgical procedures and postoperative timepoints. Across studies, pulmonary function demonstrated its greatest decline during the early postoperative period, followed by gradual improvement during subsequent follow-up. However, pulmonary function generally remained below preoperative values, with the magnitude of decline influenced by the extent of lung tissue removed. Patients undergoing lobectomy or pneumonectomy generally experienced greater postoperative reductions than those undergoing sublobar procedures, although outcomes also varied according to the lobe and number of segments resected. Patient-reported outcomes, including dyspnea, pain, physical function, and quality of life, similarly worsened during early recovery but generally improved over time, with some measures returning to baseline. Preoperative pulmonary function, particularly DLCO, remains important for assessing a patient's ability to tolerate resection, while postoperative oxygen requirements are influenced by pulmonary function, patient characteristics, and postoperative complications. Additionally, decreased physical activity after surgery was associated with non-recovery of DLCO. Overall, postoperative pulmonary function follows a pattern of early decline followed by gradual recovery, although complete return to baseline is uncommon. These findings emphasize the importance of evaluating both pulmonary function and patient-reported outcomes when considering the consequences of pulmonary resection.

## Introduction

Lung cancer is one of the most common and deadly cancers worldwide (1). Pulmonary resection remains a crucial curative treatment for patients with resectable non-small cell lung cancer (NSCLC) (2). The extent of resection is determined by the location and characteristics of the tumor and the patient's ability to tolerate loss of lung tissue (2, 3). Surgical approaches range from sublobar resection, including wedge resection and segmentectomy, to lobectomy, with the most extensive procedure being pneumonectomy (3). The scope of sublobar resection has expanded in recent years, with randomized trials supporting segmentectomy as a non-inferior option compared to lobectomy for patients with small, peripheral, node-negative NSCLC (4, 5).

Pulmonary function testing (PFT) provides a measure of pulmonary reserve before and after lung resection (2, 6). Preoperative PFT values, including forced expiratory volume in one second (FEV1), forced vital capacity (FVC), and diffusing capacity for carbon monoxide (DLCO), are used to determine baseline function and surgical risk (2). Following resection, PFTs have been shown to quantify the impact of surgery. In general, pulmonary function declines immediately postoperatively before partially recovering, with greater reductions following more extensive resections (6). These changes in postoperative PFTs may have important consequences for patients, including dyspnea and persistent respiratory symptoms. Additionally, following lung resection, patients may report pain, fatigue, and reduced physical functioning, especially during early recovery (6, 7).

Despite the widespread use of PFTs in evaluating patients before and after pulmonary resection, the measured decline can vary substantially across studies. Differences in patient populations, baseline pulmonary function, and extent of resection likely contribute to this variability (6, 8). It has been found that postoperative changes in PFTs are associated with patient-reported outcomes, therefore PFTs alone may not fully explain the impact of resection on recovery (6, 9). In this narrative review, we will investigate PFTs and patient reported outcomes after lung resection to determine the clinical implications of these postoperative changes.

## Patient outcomes before and after surgical resection

### Quality of life

Changes in postoperative pulmonary function can have a vast impact on patients' quality of life. Quality of life after pulmonary resection is not only based on how much tissue is removed; it also depends on the symptoms patients experience postoperatively. Balduyck et al. found that lobectomy and wedge resection patients had a temporary decline in quality of life measures, including increased pain at 1 month, which all returned to baseline by 3 months. While dyspnea increased in patients who underwent lobectomy, it was not significantly different than those who underwent wedge resection. Those receiving pneumonectomy did not return to baseline during the 12-month follow-up period in regards to physical function, pain, and dyspnea (7). Similar findings were reported in a prospective study comparing lobectomy and pneumonectomy. At six months, the physical function decline was significantly greater after pneumonectomy than after lobectomy, while mental health scores did not differ significantly (10). Sublobar resection may also have some advantages in terms of postoperative symptoms. A retrospective study comparing sublobar resection and lobectomy found that both groups initially experienced a decline in quality of life, but patients undergoing sublobar resection reported fewer complications regarding chest tightness, shortness of breath, breathlessness, cough, and phlegm (11). Shin et al. measured quality of life through the mMPC dyspnea score and CAT score in patients receiving bilobectomy/pneumonectomy, lobectomy, and wedge resection/segmentectomy. These scores were initially worse in the bilobectomy/pneumonectomy group, but this difference resolved by 6 months for the CAT score and by 1 year for the mMPC dyspnea scale. For all surgery types, the CAT score even returned to baseline by 1 year (6). These studies suggest that while a smaller resection may lead to fewer or less persistent quality of life deficits, these seem to have decent recovery with time.

### Supplemental oxygen

Supplemental oxygen is another important consideration after pulmonary resection, particularly in patients with poor baseline pulmonary function. A 2024 systematic review including more than 26,000 patients found that approximately 14.5% were discharged with new home oxygen after pulmonary resection (12). However, the factors associated with postoperative oxygen requirement varied across studies. Nicastri et al. found that patients undergoing both sublobar resection and pneumonectomy had higher odds of requiring home oxygen compared with those undergoing lobectomy (13), indicating that the association between extent of resection and postoperative oxygen requirement may be more complex than expected. In contrast, other studies identified patient and physiologic characteristics as important predictors (14–16). Lower preoperative FEV1 was associated with postoperative home oxygen requirement in several studies, although this association was not consistent after multivariable adjustment (12, 14, 15). Additionally, decreased preoperative DLCO was associated with postoperative oxygen requirements in univariate analyses (14, 16, 17). Nicastri et al. found that in all surgery types, a DLCO below 60% was associated with increased odds of requiring home oxygen, and a DLCO below 40% for sublobar resections specifically was a significant predictor for home oxygen (17). Other reported risk factors included exercise-induced hypoxia, obesity, and postoperative pulmonary complications (13, 16, 17). These findings suggest that postoperative oxygen requirements are influenced by multiple patient and procedure related factors, rather than being determined by resection extent alone.

## Pulmonary function testing before and after surgical resection

### Preoperative and postoperative comparisons within surgical resection type

The type of lung resection a patient receives depends on the general factors of where the tumor lies and its dimensions. If a wedge resection can be performed with successful margins, it is preferred because it preserves the most lung tissue. Gu et al. investigated the postoperative changes in PFTs across lung resections. For wedge resections, at 6 months, the decline of PFTs was reported as 5.5%, 8.6%, and 11.2% for FVC, FEV1, and DLCO, respectively (18). Another study reported their postoperative PFTs for wedge resections at 12 months and saw a decline of 8%, 9%, and 7% for FVC, FEV1, and DLCO, respectively (19). While a direct comparison cannot be made between the two studies, it does show that at different timepoints during recovery the PFTs can improve.

Segmentectomies are the second best at preserving lung tissue while obtaining margins. Instead of only taking a small amount tissue surrounding the tumor, like a wedge, this procedure removes the entire lung segment containing the tumor. Gu et al. reported outcomes at 6 months and showed a decline of 15%, 18.4%, and 13.5% for FVC, FEV1, and DLCO, respectively (18). Bao et al. separated their segmentectomy procedures into single vs. multiple for comparison to lobectomies. Preoperatively, the multiple segmentectomy group had better FVC and FEV1 measurements compared to the single segmentectomy cohort, but significance between these two was not reported. They investigated the postoperative PFTs at 12 months, finding the single segmentectomy cohort had significantly better outcomes, meaning a smaller decline in PFTs, than the multiple segmentectomy cohort. Overall, both groups had a combined range of 4%–9% and 5%–10% decline in FVC and FEV1, respectively (20). Handa et al. separated their segmentectomies into simple and complex procedures, with complex referring to a surgery requiring the creation of several intersegmental planes and a fear that postoperative recovery would be negatively affected. At 12 months, there was no significant difference between the PFTs for either surgery, which held true when comparing the number of segments resected as well. They reported a decline of 9%–10%, 9%–10%, and 7%–10% for FVC, FEV1, and DLCO, respectively (19). Comparing the study reporting outcomes at 6 months to the ones reporting them at 12 months, it again shows that PFTs gradually improve during follow-up.

Lobectomies are the second most extensive resection. Brunelli et al. investigated the outcomes of 180 patients undergoing lobectomy and found that while patients had a significant decline between their preoperative and postoperative PFTs, they had significant increases during follow-up at both 1 and 3 months. However, this 3-month value was still significantly lower than the preoperative measurement (21). One study investigated outcomes following lobectomy at three different points during follow-up. At two weeks, the decline in PFTs reached 23.3% for FVC and 21.7% for FEV1. These values improved to −12.3% and −8.5% at 6 and 12 months for FVC, respectively and to −11.6% and −8.4% at 6 and 12 months for FEV1, respectively. In addition to PFTs, they investigated patient-reported outcomes using the mMRC dyspnea scale and CAT score. This showed that in addition to PFTs improving, patient-reported outcomes also improved with time, with the CAT score having full recovery (6). Jeon et al. also investigated outcomes of lobectomies at different timepoints during follow-up to see if the lobe resected affected postoperative PFTs. For all resections, the steepest decline was at the 2-week follow-up, with improvement shown at 12 months. Using the predicted postoperative PFTs, they found that resecting the right upper lobe (RUL) had the worst outcomes, reporting non-recovery of PFTs at 6 months was more likely in this cohort. In all lobe-resection groups, a large decrease in physical activity reported at their 2-week follow-up was associated with non-recovery of DLCO. Also, when comparing the actual to the predicted postoperative measurement, those who had substantial declines past the prediction had increased postoperative complications (22). These studies show that PFTs and symptoms improve with time; however, a full recovery is rarely made and the specific lobe resected might affect these outcomes.

Finally, pneumonectomies represent the most extensive resection. Postoperatively, one study showed that after the significant decline of PFTs, the FEV1 improved during follow-up at 1- and 3 months, but not significantly. Additionally, the measured DLCO did significantly improve from discharge to 1-month, but not between 1- and 3-month follow-up. For both FEV1 and DLCO, there continued to be a significant difference from PFTs measured before surgery (21). An older study also investigated outcomes following pneumonectomy and included PFTs, gas exchange, and exercise capacity at different timepoints. They found that all three variables were significantly decreased at 3 months with slight recovery by 6 months, but not to preoperative values. Furthermore, regarding factors limiting exercise, the pneumectomy cohort had significantly decreased breathing reserve at 3 months that improved by 6 months, but not significantly. This group also reported significantly more dyspnea and muscle fatigue as reasons for stopping exercise at 3 months, but this decreased significantly by 6 months (23). As pneumonectomy removes the most amount of lung tissue, it is understandable that a significant decline in postoperative PFTs and decreased exercise capacity without full recovery accompany this surgery.

### Preoperative and postoperative comparisons between surgical resection type

Multiple studies have made comparisons within their cohorts of patients who underwent different surgical resections. A study of 620 patients compared bilobectomy/pneumonectomy, lobectomy, and wedge resection/segmentectomy with follow-up at 2 weeks, 6 months, and 1 year. They found that across procedures, PFTs dramatically decreased at the 2-week follow-up, then steadily improved afterwards, but never back to baseline. In general, the PFTs were worse in procedures that resected more lung tissue during all follow-up timepoints (6). Another study compared pneumonectomy to what they termed as lobectomy, but lobectomy in this study meant anything less than pneumonectomy with segmentectomies and wedge resections grouped in. They included measurements of PFTs, gas exchange, and exercise tolerance at their three follow-ups and compared outcomes between the two groups. They reported a significant decrease in all parameters at both 3 and 6 months for the pneumonectomy cohort. Furthermore, in the pneumectomy group, breathing reserve was significantly decreased and subjective reports of dyspnea and muscle fatigue stopping exercise were significantly increased compared to the lobectomy cohort (23). While the inclusion of objective and subjective factors affecting outcomes makes this study interesting to note, the broad definition of lobectomy makes it difficult to truly draw conclusions besides pneumonectomy having poorer outcomes.

A large study of 1,284 patients undergoing single segmentectomy (SSE), multiple segmentectomy (MSE), and lobectomy compared outcomes at 12 months. Interestingly, preoperative PFTs were significantly better in the MSE cohort. At 12 months, the segmentectomy groups had significantly better PFTs than the lobectomy group, and the SSE group fared better than the MSE group. PFT loss per lung segment removed was similar across surgeries. Upon further analysis into which lobe was removed, they found that when the left upper lobe (LUL) or right lower lobe (RLL) was resected, there was no longer a PFT benefit to MSE when compared to lobectomy. Furthermore, when the left lower lobe (LLL) was resected, MSE had significantly worse PFTs when compared to lobectomy (20). A separate study investigating lobectomy vs. segmentectomy outcomes at different timepoints reported that patients who did not have PFT recovery by 12 months were more likely those receiving MSE or lobectomy. These non-recovery patients also had lower respiratory muscle strength and a greater initial decline in postoperative PFTs. Non-recovery of vital capacity specifically had worse overall survival, while the type of surgery did not explicitly affect prognosis (24).

Two studies compared outcomes between patients receiving lobectomy, segmentectomy, and wedge resection. One study reported at 6 months, FVC loss was significantly worse in lobectomy compared to segmentectomy and wedge, and in segmentectomy compared to wedge. Furthermore, FEV1 loss was significantly worse in lobectomy compared to segmentectomy and wedge, but no significant difference was found between the segmentectomy and wedge cohorts. Interestingly, they described an increased loss of PFT per segment removed when comparing segmentectomy and lobectomy (18). This difference could possibly be explained by the way they performed the segmentectomies leading to inadequate lung re-expansion. Handa et al. initially compared complex and simple segmentectomies, but after finding no significant differences in outcomes, they only used complex segmentectomies to compare to lobectomy and wedge resection. Interestingly, preoperative measures of DLCO significantly differed between the three surgeries because wedge resection had lower measurements. At 12 months, PFTs significantly differed between complex segmentectomy and lobectomy, with lobectomy having poorer values. While wedge resection had better PFTs, no significant difference was found between it and complex segmentectomy. Significance was not reported between wedge resection and lobectomy specifically, but wedge resection had better outcomes (19). As expected, lobectomies have worse outcomes when compared to smaller resection procedures; however, wedge resections and segmentectomies seem to have similar results across studies.

## Discussion

Lung resection for resectable NSCLC is a mainstay of surgical treatment. While the tumor characteristics make the decision of which resection type is best, knowing how patients are affected postoperatively is essential. After any lung resection, the general trend is an immediate decline in PFTs, the nadir presenting within the first couple of weeks postoperatively, then a steady incline that does not reach full recovery. Conversely, patient-reported outcomes after surgery also initially decline, but these might return to baseline over time, depending on the surgery. Prior to any lung resection surgery, preoperative PFTs, especially a measurement of the DLCO, is of utmost importance to determine a patient's ability to undergo resection (2). If patients do not have a reserve of lung function to sustain them postoperatively, the surgery should not be performed.

While functional capacity of the remaining lung tissue significant, patient-reported outcomes are just as important. In regards to pain and physical function, some studies found differences between surgeries on how quickly patients reportedly returned to their baseline (6, 7), with one study specifically highlighting mental health scores as not significantly differing (10). This finding could be for various reasons, but it's possible that patients receiving a more extensive surgery naturally had more extensive disease, so even though their physical activity is diminished, there is comfort in having a resection to remove cancer even when it's extensive. Overall, it is expected that a larger surgical resection would lead to decreased physical activity and furthermore decreased perception of quality of life, but these measures seem to improve the further from surgery.

Requiring home supplemental oxygen after surgery is another factor that must be considered prior to resection. While PFTs alone can help assess this likelihood preoperatively, there is evidence to indicate a more holistic approach should be used. Postoperative management of lung resection patients must include gradually increasing physical activity soon after surgery. Decreased physical activity after lung surgery can lead to many complications, such as thromboses, pneumonia, and prolonged hospital stays (25). One study showed that a reported decrease in physical activity after lung resection was associated with non-recovery of DLCO (22). As these patients are already losing lung tissue from their procedure, the remaining tissue must be optimized. In order to obtain the best results, encouraging patients to have daily physical activity after surgery is crucial.

While no long-term studies were found, studies that investigated outcomes at different timepoints throughout follow-up showed a steady recovery of PFTs. Within surgical subtypes, results across studies regarding PFT decline were similar when values were reported at the same follow-up. When looking at different timepoints, a trend is shown between studies of PFTs incrementally improving as you move further from surgery. Some studies had multiple follow-ups, and they were able to demonstrate improvement, some significantly, within their cohorts as time went on (6, 21–23). Many factors can be the cause of a dramatic drop in PFTs postoperatively in addition to the removal of lung tissue. For example, postoperative pain can limit a person's ability to perform PFTs effectively, possibly leading to exaggerated results early on. Furthermore, as mentioned by one study (18), the manner of resection may impact the ability of lung tissue to fully re-expand. This would lead to ineffective use of viable lung tissue postoperatively and might affect the PFT results.

Between surgical subtypes, it is unsurprising that the more lung tissue removed correlates with poorer PFTs postoperatively. Two studies comparing extent of surgical resection reported differences in preoperative PFTs, with the more extensive resection groups having better measurements (19, 20). Whether or not the preoperative PFTs influenced the extent of surgical was not discussed, but it is interesting to consider that patients with better preoperative pulmonary function might be able to receive more extensive resections if a choice is available. An interesting theme investigated by some studies was whether the lung section removed has effects on postoperative outcomes. Two studies discovered significant changes depending on which lobe was removed, specifically that resecting the RUL has poorer outcomes (20, 22). As the RUL has been found to be the main site of lung cancer (26), knowing removal of the entire lobe might worsen outcomes is important for surgical consideration. One study also showed segmentectomy of the LLL resulted in significantly worse postoperative PFTs compared to lobectomy (20). As segmentectomy removes less tissue, this finding directly contradicts the idea that more tissue removed leads to worse PFTs. However, the cohort undergoing LLL resection specifically was quite small, so this finding might not truly represent universal outcomes.

A recent study investigated the postoperative complications in a cohort of patients undergoing lung resection. They found that advanced age and male sex were significantly associated with postoperative complications, similar to other studies. Also, comorbidities such as COPD, coronary disease, and dyslipidemia were significantly associated with postoperative complications (27). Therefore, patients with these known risk factors must be optimized prior to and after surgery with items such as preoperative pulmonary rehabilitation and early postoperative physical activity.

As this is a narrative review, no formal selection criteria were used in our literature search. Therefore, inherent limitations to this review exist, such as the possibility that studies were missed and the potential for selection bias. To mitigate this risk, we tried to keep our search criteria broad in order to capture multiple papers, as well as include mainly primary sources regarding this topic. Furthermore, the initial decline in PFTs reported by studies can be due to a multitude of factors relating to the surgery itself rather than true loss of functional lung tissue. As patients can be in significant pain or have any number of postoperative complications that inherently reduce the functionality of lung parenchyma, it may be difficult to truly attribute an initial decline to the loss of tissue rather than a normal postoperative course.

Awareness of postoperative PFTs and patient-reported outcomes allows for an informed decision into which surgery is best. While longer-term studies are required, current studies show that postoperative PFTs improve with time and patient-reported outcomes might even return to baseline. Even though the tumor characteristics supersede other factors regarding which surgical treatment is chosen, when more extensive resections are required, being able to discuss the possible outcomes with patients is imperative.
